# Lithium phosphonate umpolung catalysts: Do fluoro substituents increase the catalytic activity?

**DOI:** 10.3762/bjoc.7.138

**Published:** 2011-08-31

**Authors:** Anca Gliga, Bernd Goldfuss, Jörg M Neudörfl

**Affiliations:** 1 Department für Chemie, Universität zu Köln, Greinstr. 4, D-50939 Köln, Germany

**Keywords:** benzoin coupling, catalytic activity, fluoro effects, lithium phosphonates, umpolung

## Abstract

Fluorinated and nonfluorinated phosphonates are employed as precatalysts in lithium phosphonate catalyzed cross benzoin couplings. Surprisingly, a decreased catalytic activity for the fluorinated precatalysts compared to the nonfluorinated systems is observed. Furthermore, the ring size of six, seven and nine membered ring catalysts appears not to be crucial for their catalytic activity.

## Introduction

**Scheme 1:**
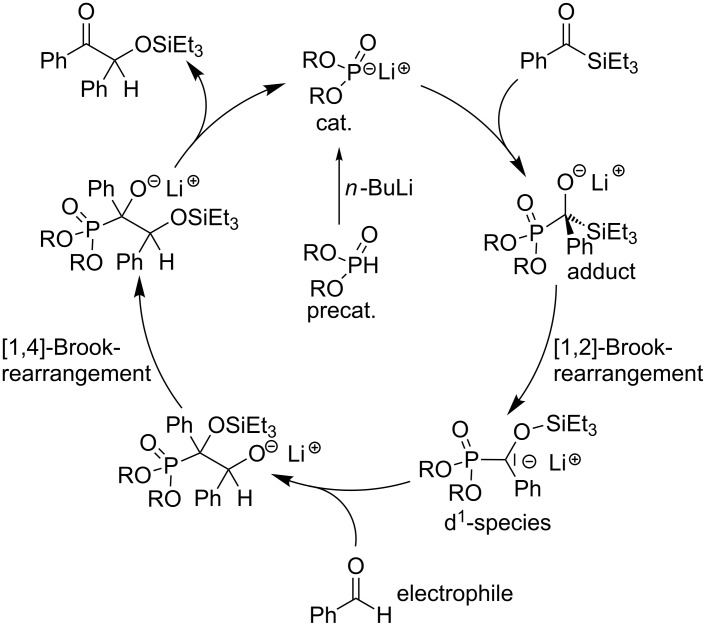
Proposed catalytic cycle of the lithium phosphonate catalyzed cross benzoin coupling [18].

Since the discovery of the cyanide catalyzed benzoin reaction by Liebig and Wöhler in 1832 [1], acyloin-type reactions evolved as powerful tools for couplings of acylanion equivalents with carbon electrophiles. In addition to cyanide [2–5] and nucleophilic carbene catalysts (e.g. thiazolium salts) [6–17], lithium phosphonates were found to catalyze cross acyloin type couplings of acylsilanes with aldehydes [18]. The catalytic cycle proposed by Johnson et al. [18] (Scheme 1) suggests that a potential metallophosphonate catalyst must act as a nucleophile, an anion (d^1^-synthon) stabilization group, and as well as a leaving group (nucleofuge). Comparative computational assessments of carbanionic d^1^-species, which have been proposed as crucial intermediates according to the Lapworth and Breslow mechanisms, show comparable activities for lithium phosphonate and cyanide [19–20].

**Scheme 2:**
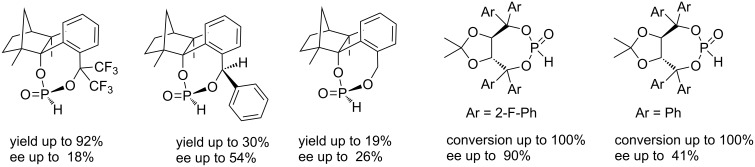
Phosphonate as precatalysts in benzoin coupling [18,26].

Recently, we introduced fenchol based phosphonates as precatalysts, which are similarly accessible as fencholate metal catalysts [21–25], in the benzoin coupling (Scheme 2) [26]. A strong increase of the catalytic activity was observed for a benzylic fencholate, when the benzylic positions were occupied by CF_3_-groups (92% versus 19% yield, Scheme 2) [26]. This increased reactivity is thought to arise from a favored formation of the carbanionic d^1^-synthon intermediate, due to the electron withdrawing effect of the CF_3_ groups. A comparison of fluorinated and nonfluorinated TADDOL phosphonates (which were used by Johnson's group) as precatalysts in benzoin coupling does not show any difference in reactivity (Scheme 2). In contrast the enantioselectivity is clearly higher with the fluorinated TADDOL precatalyst (Scheme 2).

Here, we analyze the effect of fluoro substituents on the catalytic activity by using different fluorinated and nonfluorinated phosphonates as precatalysts in the benzoin coupling.

## Results and Discussion

**Scheme 3:**
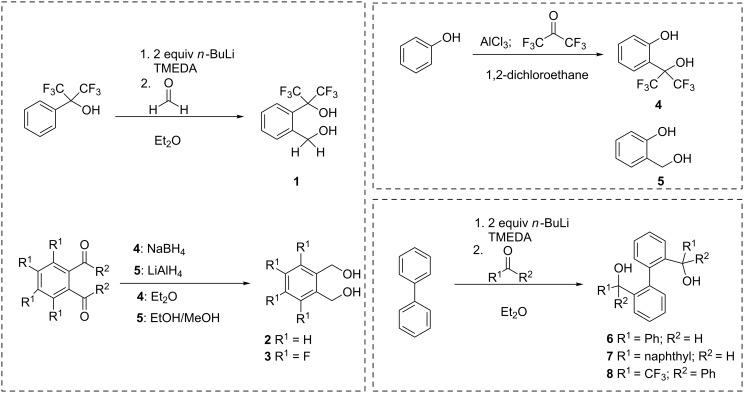
Synthesis of diols **1–4**, **6**–**7**; diol **5** is commercially available.

As precursors for six, seven and nine membered ring phosphonates, diols **1**–**4**, and **6**–**8** were synthesized (Scheme 3). The synthesis of diol **1** was conducted by an *ortho* lithiation of 1,1,1,3,3,3-hexafluoro-2-phenylpropan-2-ol and subsequent addition of the in situ generated carbanion to formaldehyde. For comparison, a nonfluorinated diol precursor **2** was synthesized. Diol **3** was used as the precursor to investigate the influence of aromatic fluoro substituents. Six ring phosphonates were realized with diol **4** [27] and the analogous nonfluorinated 2-(hydroxymethyl)phenol (**5**) as precursor.

**Figure 1:**
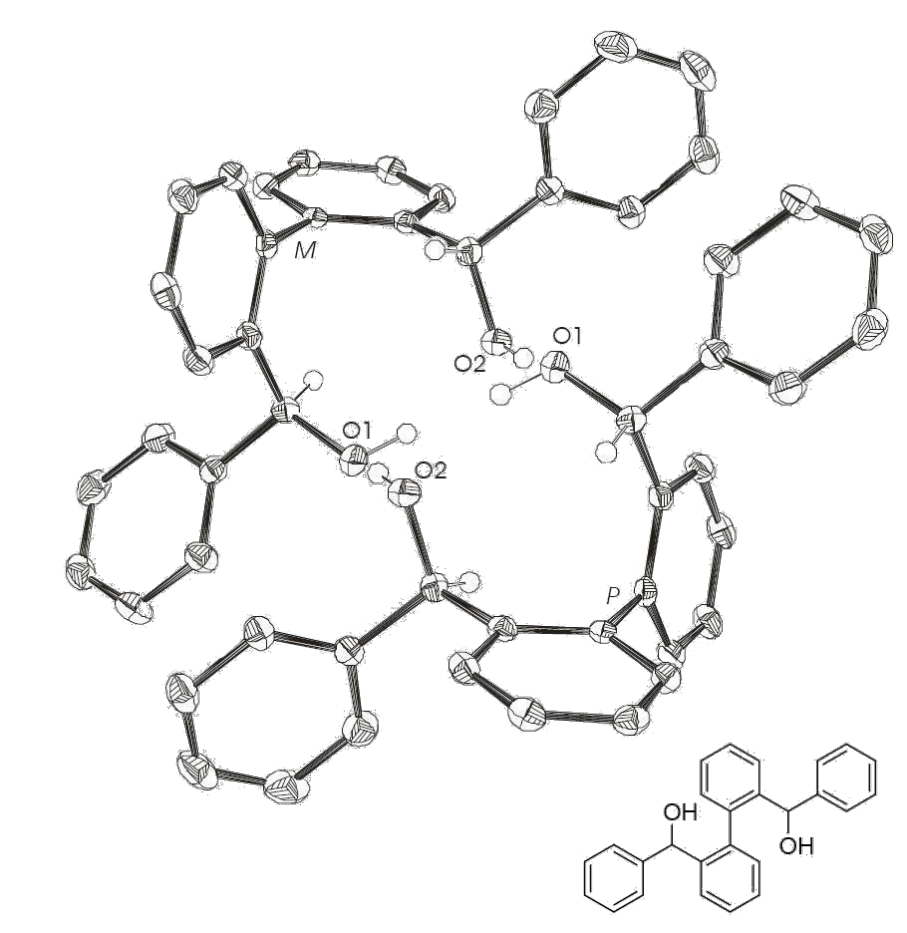
X-ray crystal structure of **6**. (*M*)*-*(*S,S*) and (*P*)*-*(*R,R*) pair of enantiomers; intermolecular O1–O2 distance 2.81 Å; intramolecular O1–O2 distance 2.83 Å. Ellipsoids correspond to 50% probability levels. Hydrogen atoms are omitted for clarity.

**Figure 2:**
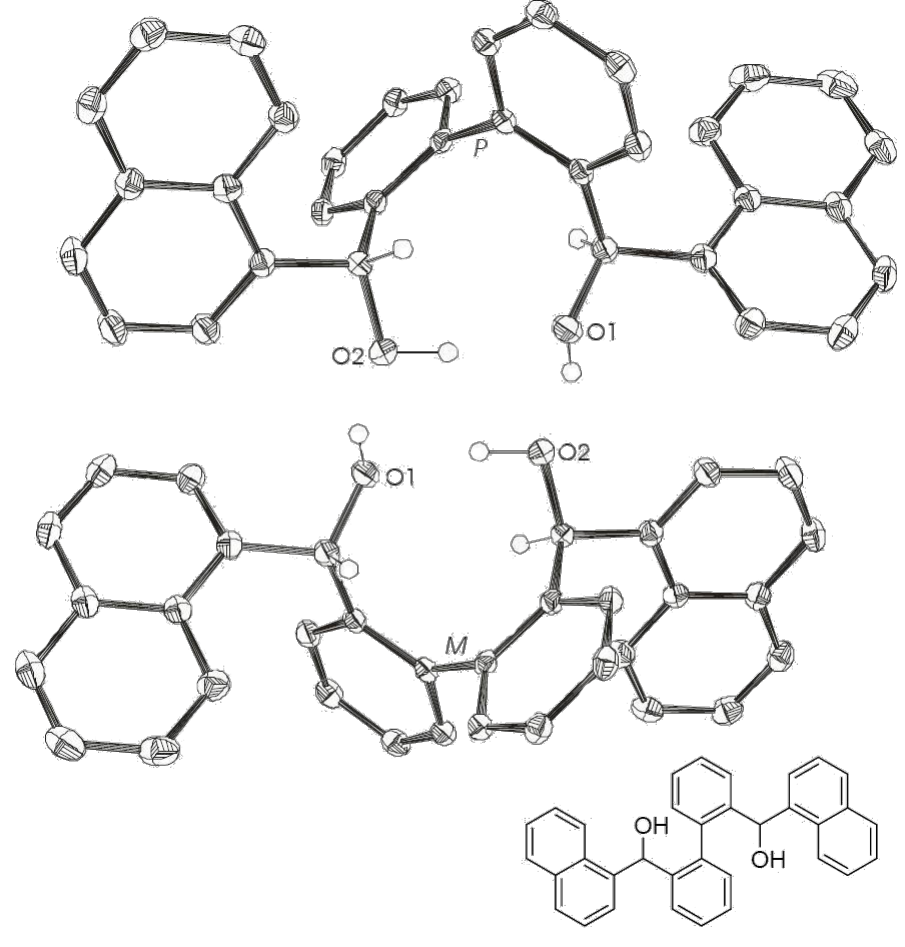
X-ray crystal structure of **7**. (*M*)*-*(*S,S*) and (*P*)*-*(*R,R*) pair of enantiomers; intermolecular O1–O2 distance 2.80 Å; intramolecular O1–O2 distance 2.81 Å. Ellipsoids correspond to 50% probability levels. Hydrogen atoms are omitted for clarity.

Biphenyl-based fluorinated and nonfluorinated systems (**6** [28–30], **7** and **8**, Scheme 3) were chosen as precursors for the synthesis of nine ring phosphonates. The synthesis of these diols was realized by a double *ortho* lithiation of biphenyl and subsequent addition to the corresponding carbonyl compound. By this procedure, two asymmetric carbon centres and a chiral axis, which is fixed by intramolecular hydrogen bonds (**6**: Intramolecular O1–O2 distance 2.83 Å, **7**: Intramolecular O1–O2 distance 2.81 Å, Figure 1 and Figure 2, respectively), are generated. Chiral HPLC and X-ray analyses revealed one pair of enantiomers for diol **6** and **7** (HPLC (Daicel-OD-H, 90:10 *n*-hexane/isopropanol; flow 0.5 mL/min): **6**: *t*_R1_ = 10.4 min; *t*_R2_ = 13.2 min (racemate); **7**: *t*_R_ = 22.1 min; X-ray structures shown in Figure 1 and Figure 2, respectively) and an additional *meso* product for diol **8** (HPLC: *t*_R2_ = 22.4 min; *t*_R3_ = 30.6 min (racemate); *t*_R1_ = 9.1 min). A dimer associated by a hydrogen bond is apparent for the enantiomeric pair (**6**: intermolecular O1–O2 distance 2.81 Å, **7**: intermolecular O1–O2 distance 2.80 Å, Figure 1 and Figure 2, respectively). The favored formed enantiomeric pair has the same configuration (*R*,*R* or *S*,*S*) for both benzylic carbon centres, which defines the conformation of the biphenyl axis (*M*(*inus*) for *S*,*S* and *P*(*lus*) for *R*,*R*).

The conformational stability of the biphenyl axis can be demonstrated by the energy difference of the optimized structures (B3LYP 6-31G*) (Table 1) (between (*M*)*-*(*S,S*)*;* (*P*)*-*(*R,R*) and (*P*)*-*(*S,S*)*;* (*M*)*-*(*R,R*) for **6** *E*_rel_ = 3.6 kcal/mol; for **7** *E*_rel_ = 4.3 kcal/mol). The alternative diastereomers, with different configurations at the benzylic carbon (*M*)*-*(*R,S*)*;* (*P*)*-*(*R,S*), are energetically disfavored (Table 1). For these diastereomers two possible directions of the hydrogen bond were considered, that is (*M*)*-*(*R,S*)*;* (*P*)*-*(*R,S*) with the hydrogen bond from *S*→*R* giving *E*_rel_ = 1.4 kcal/mol and (*M*)*-*(*R,S*)*;* (*P*)*-*(*R,S*) with the hydrogen bond from *R*→*S* giving *E*_rel_ = 1.8 kcal/mol for **6**; equivalently *E*_rel_ = 4.7 kcal/mol and *E*_rel_ = 4.4 kcal/mol in the respective cases for **7**. Similar biphenyl conformation stabilities were found for 1,1′-biphenyl-2,2′-bisterpenols (terpenol moiety: (−)-Fenchol, (−)-menthol, (−)-verbenol und (−)-carvol) [31–32]. The energy differences of the terpene-based conformers are between 5.1 and 5.8 kcal/mol [32–33] (B3LYP/6-31++G**:AM1).

**Table 1:** Optimized structures of diols **6** and **7**.

	diol **6**			

diastereomer	(*M*)*-*(*S,S*)*;* (*P*)*-*(*R,R*)	(*P*)*-*(*S,S*)*;* (*M*)*-*(*R,R*)	(*M*)*-*(*R,S*)*;* (*P*)*-*(*R,S*) *R*→*S*^a^	(*M*)*-*(*R,S*)*;* (*P*)*-*(*R,S*) *S*→*R*^a^
*E*_rel_ [kcal/mol]^b^	0	+3.6	+1.8	+1.4

	diol **7**			

diastereomer	(*M*)*-*(*S,S*)*;* (*P*)*-*(*R,R*)	(*P*)*-*(*S,S*)*;* (*M*)*-*(*R,R*)	(*M*)*-*(*R,S*)*;* (*P*)*-*(*R,S*) *R*→*S*^a^	*M*)*-*(*R,S*)*;* (*P*)*-*(*R,S*) *S*→*R*^a^
*E*_rel_ [kcal/mol]^b^	0	+4.3	+4.4	+4.7

^a^Direction of hydrogen bond; ^b^B3LYP 6-31G*.

**Scheme 4:**
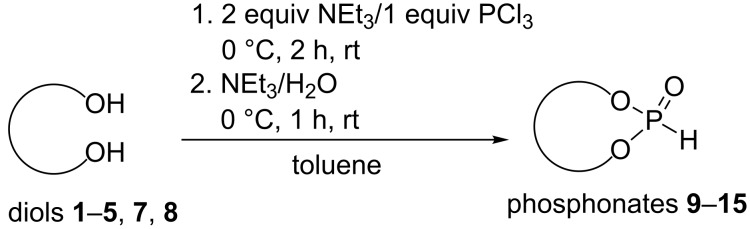
Synthesis of phosphonates **9**–**15**.

The conversion of diols **1**–**5**, **7**, and **8** to the desired phosphonates can be achieved by twofold addition to phosphorus trichloride and subsequent hydrolysis. Diol **6** could not be converted under the employed conditions (Scheme 4).

The strong inductive effect of the fluoro substituents is clearly visible from the ^1^*J*(P–H) coupling constants, which are significantly increased compared to the nonfluoro-substituted phosphonates (Table 2). In general, ^1^*J*(P–H) coupling constants increase with the electronegativity of the substituents [33]. The influence of electronegativity results from the change in s-character, given that the Fermi-contact is the dominant coupling mechanism [33]. According to Bent’s rule [34] electron withdrawing substituents require more p-character in the bonding orbitals, which leads to an increased s-character in the bonding P–H orbital. The smallest influence on the coupling constant is apparent for phosphonate **11**, in which the fluoro substituents are not in close proximity to the phosphorous atom (five bonds distance). The largest ^1^*J*(P–H) coupling constant was detected for phosphonate **13**. Thereby an additional electron withdrawing effect of the phenoxy group causes the further increase in the ^1^*J*(P–H) coupling constant. All synthesized phosphonates were identified by ^31^P NMR, especially characteristic are the phosphorus–hydrogen and phosphorus–fluoro couplings.

**Table 2:** ^1^*J*(P–H) coupling constants for phosphonates **9**–**15**.

	seven membered ring			

phosphonate	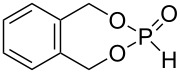 **9**	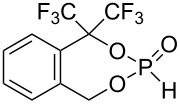 **10**		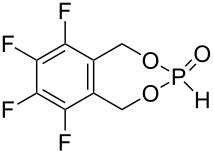 **11**
^1^*J*(P–H) [Hz]	705.3	755.0		720.0

	six membered ring			

phosphonate	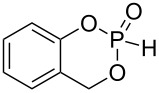 **12**		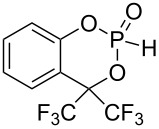 **13**	
^1^*J*(P–H) [Hz]	723.0		758.0	

	nine membered ring			

phosphonate	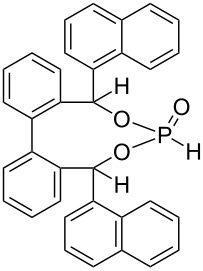 **14**		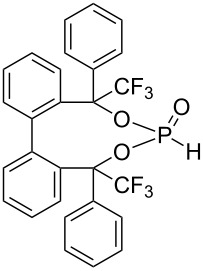 **15**	
^1^*J*(P–H) [Hz]	713.2		754.7	

**Figure 3:**
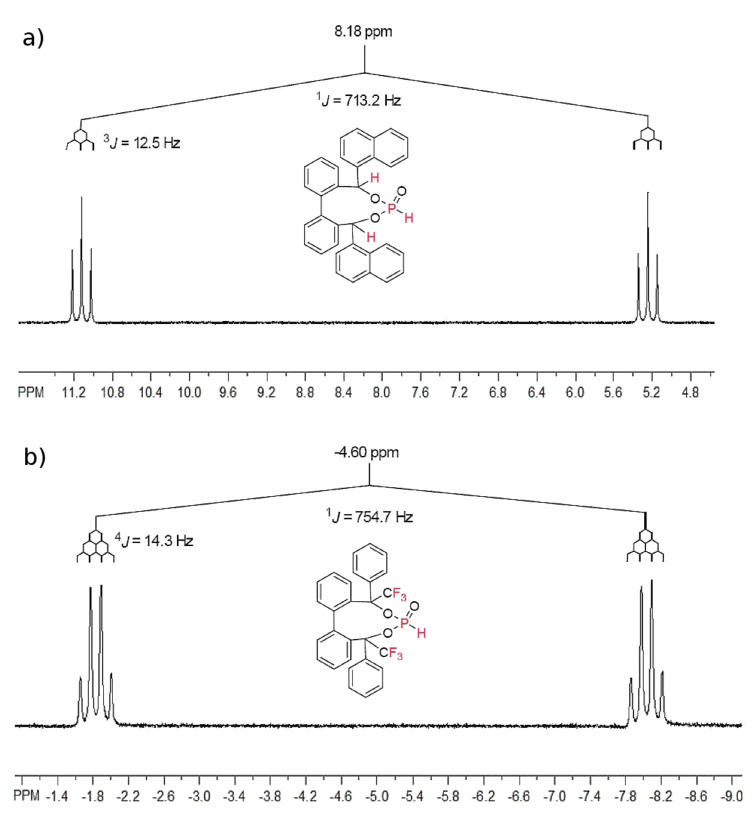
^31^P NMR of phosphonate **14** (a) and **15** (b).

For phosphonates **14** and **15** (Figure 3) a doublet splitting caused by the ^1^*J*(P–H) coupling ~700 Hz was observed in the ^31^P NMR spectra. The protons in the benzylic position (phosphonate **14**) effect a ^3^*J*(P–H) coupling (12.5 Hz) and the CF_3_ groups in this position (phosphonate **15**) a ^4^*J*(P–F) coupling (14.3 Hz) (Figure 3).

**Figure 4:**
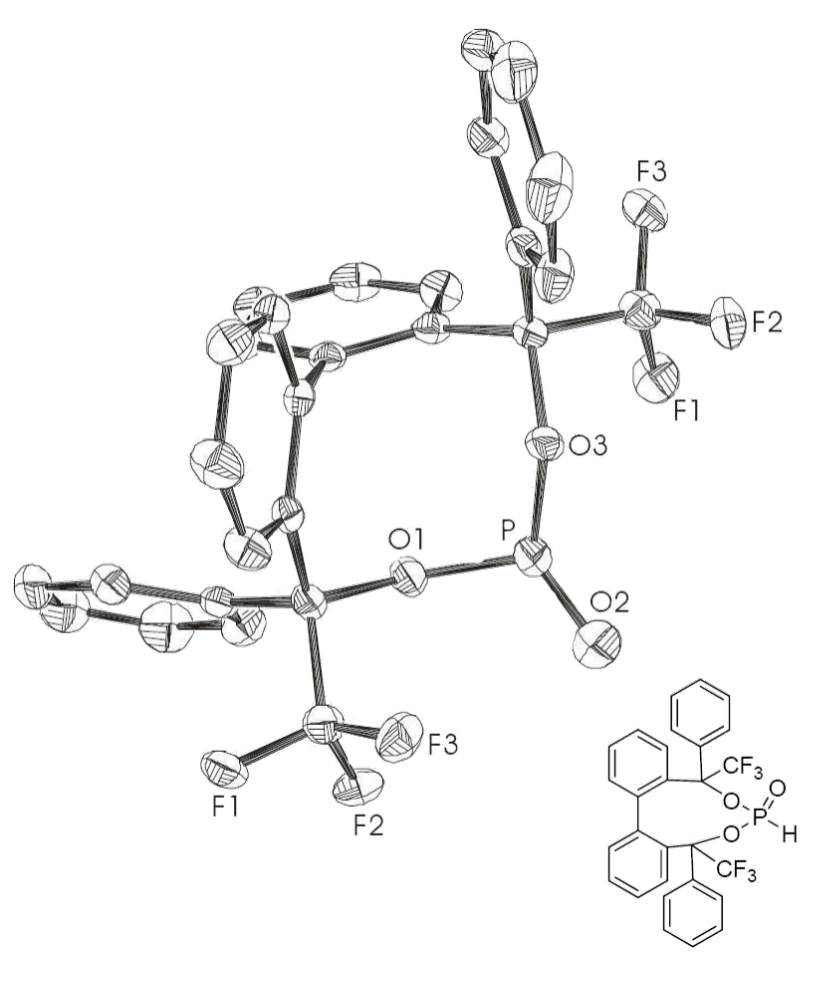
X-ray crystal structure of **15**. (*M*)*-*(*R,S*) diastereomer; ellipsoids correspond to 50% probability levels. Hydrogen atoms are omitted for clarity.

A crystal structure was obtained for phosphonate **15**, which shows the (*M*)*-*(*R,S*) diastereomers (Figure 4).

**Scheme 5:**
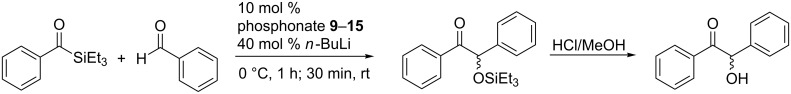
Lithium phosphonate catalysts in cross benzoin coupling.

**Figure 5:**
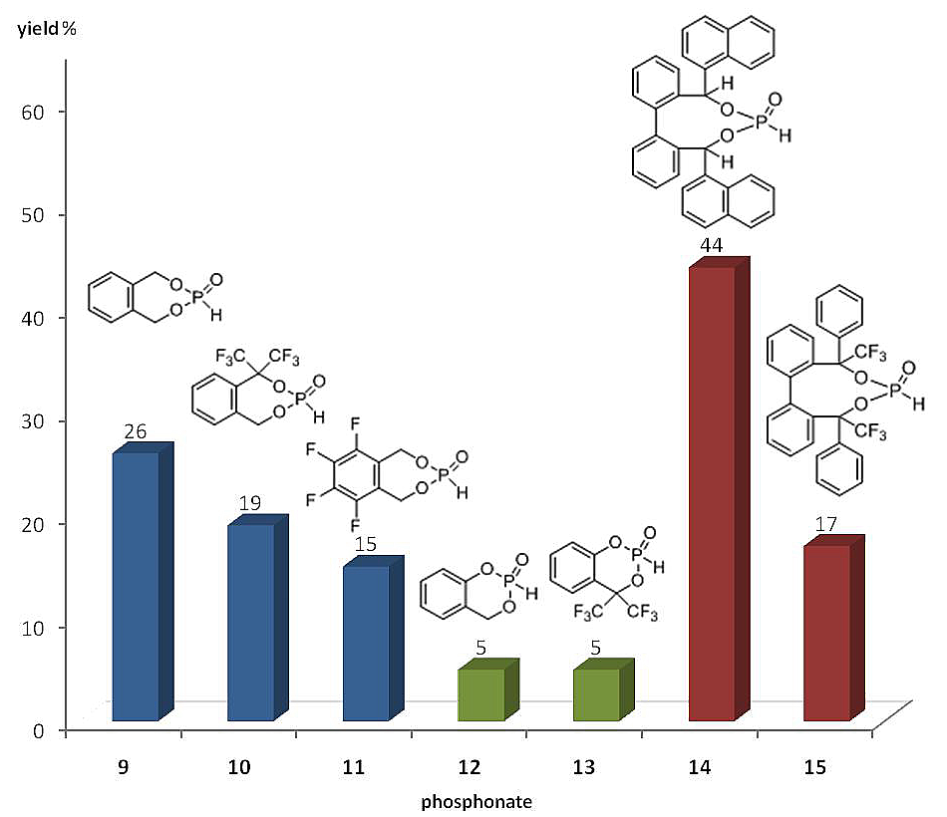
Phosphonate precatalysts **9**–**15** in cross benzoin coupling.

The lithium phosphonate catalyzed benzoin reaction (Scheme 5) with phosphonates **9**–**15** as precatalysts led to the benzoin product in low to moderate yields (5–44%) (Figure 5). The supposed increase in catalytic activity, which was observed for fenchol-based phosphonate (Scheme 2) [21] with fluoro-substituted phosphonates as precatalysts, could not be confirmed. The highest yield was achieved with phosphonate **14** as precatalyst (44%). Contrary to expectations this yield is twice as high as the yield achieved with phosphonate **15**. The reduction of the nucleophilic character of the phosphorus nucleophile in the first step of the catalytic cycle, and of the d^1^-synthon in the third step of the catalytic cycle (Scheme 1), could explain these results. The increased ^1^*J*(P–H) coupling constants for the CF_3_ substituted phosphonates (**10**, **13**, **15**, Table 2) suggest an increase in s-character at the phosphorus atom, which confirms a reduction in nucleophilic character. In contrast to the fenchol-based phosphonates [26] the best result was achieved by a nine membered ring phosphonate instead of a seven membered ring phosphonate. It can be concluded that the ring size is not of basic importance to the catalytic activity.

## Conclusion

Three types of cyclic fluorinated and nonfluorinated phosphonates were synthesized and used as precatalysts in cross benzoin couplings with yields ranging from 5 to 44%. The inductive effect of CF_3_ substituents in benzylic position of phosphonates **10**, **13** and **15** gives rise to increased ^1^*J*(P–H) couplings in P–H precatalysts and hence points to increased s-character at the phosphorus lone pair in the active anionic catalysts [33]. A rise of catalytic activity due to the inductive effect of CF_3_ substituents, as was observed before for a fenchol-based phosphonate (Scheme 2) [26], was not realized with the phosphonates employed herein. Instead a reduction of catalytic activity was apparent with fluorinated phosphonates compared to the nonfluorinated phosphonates. This can be explained by a weaker nucleophilic character of the phosphorus nucleophile, as a consequence of the increased s-character. Comparisons of phosphonates with different ring sizes show that the nine ring phosphonates result in higher yields than do the six and seven ring phosphonates.

## Supporting Information

Experimental procedures, characterization data for compounds **1**–**4**, **6**–**15**, crystallographic data for compounds **6**, **7**, **15**, computational details for compounds **6**, **7** and general procedure for phosphonates as precatalysts in cross benzoin coupling.

File 1 Experimental procedure and characterization data.
