# Supplementary material for: Lithium phosphonate umpolung catalysts: Do fluoro substituents increase the catalytic activity?
Source: Beilstein J Org Chem. 2011 Aug 31;7:1189–97. doi: 10.3762/bjoc.7.138 (PMC3182427; doi:10.3762/bjoc.7.138)
Supplement: File 1 — Experimental procedure and characterization data. [file Beilstein_J_Org_Chem-07-1189-s001.pdf]

**Supporting Information**

**for**

# Lithium phosphonate umpolung catalysts: Do fluoro substituents increase the catalytic activity?

Anca Gliga, Bernd Goldfuss* and Jörg M. Neudörfl

Address: Department für Chemie, Universität zu Köln, Greinstr. 4, D-50939 Köln, Germany

Email: Bernd Goldfuss - goldfuss@uni-koeln.de

* Corresponding author

## Experimental procedure and characterization data

### General remarks

All experiments were carried out under argon by means of Schlenk techniques. Solvents were dried by standard methods and distilled freshly under argon prior to use. NMR spectra were recorded on Bruker DPX (300 MHz) and Bruker AV 300 spectrometers. GC/MS spectra were recorded on Agilent equipment model 6890 for the GC and model 5975 for the mass detector. The gas chromatograph was equipped with a HP-5MS column (Macherey-Nagel, 5% phenylmethylsiloxane, length 30 m, diameter 250 µm) and the used method was 50–300M.

**Synthesis of diol 1**

2 mL (12.0 mmol) 1,1,1,3,3,3-hexafluoro-2-phenylpropan-2-ol and 3.6 mL (24.0 mmol) *N*,*N*,*N*',*N*'-tetramethylethylenediamine (TMEDA) were diluted in 30 mL dry ether and cooled to 0 °C. To this solution 15 mL (24.0 mmol, 1.6 M in hexane) *n*-BuLi was added dropwise by syringe. The reaction was allowed to warm to rt and was stirred for 20 h. To the resulting solution formaldehyde was added over a period of ½ h and stirred at rt for an additional hour. Aqueous $NH_4Cl$ solution and brine were added. The organic phase was separated and the aqueous phase extracted three times with 20 mL $CH_2Cl_2$. The combined organic phases were dried over $MgSO_4$ and concentrated under reduced pressure. The crude product was purified by flash column chromatography on silica gel with hexane/ethylacetate (4:1) as eluent.

**Analytic and spectroscopic data for 1:** Yield 1.8 g (6.5 mmol, 54%); m.p. 73 °C; $^{1}H$ NMR (300 MHz, $CDCl_3$) δ 4.95 (s, 2H), 6.91 (d, *J* = 8.2 Hz, 1H), 7.01 (t, *J* = 7.9 Hz, 1H), 7.29–7.48 (m, 4H) ppm; $^{13}C$ NMR (75 MHz, $CDCl_3$) δ 155.6, 138.2, 132.8, 131.6, 130.3, 129.1, 124.8, 121.0, 113.5, 66.4 ppm; $^{19}F$ NMR (282.4 MHz, $CDCl_3$) δ −75.5 ppm; GC/MS: $t_R$ = 6.08 min. (EI) *m*/*z*: 274, 256, 227, 209, 187, 167, 137, 107, 89, 69, 51.

**Synthesis of diol 4**

0.05 g $AlCl_3$, (0.7 mmol) and 3.25 g (34.6 mmol) phenol were diluted in 60 mL dry 1,2-dichloroethane. After the solution was degassed it was cooled down to −35 °C and 1,1,1,3,3,3-hexafluoroacetone was added over a period of 20 min. The reaction was allowed to warm to rt and stirred for 48 h. The generated gas was removed by Argon and trapped in a washing bottle. 25 mL distilled water was added. The organic phase was separated and the aqueous phase extracted three times with 10 mL $CH_2Cl_2$. The combined organic phases were dried over $MgSO_4$ and concentrated un-

der reduced pressure. The crude product was purified by sublimation (60 °C) and recrystallized from *c*-hexane to give the diol as colorless crystals.

**Analytic and spectroscopic data for 4:** Yield 6.1 g (23.8 mmol, 67%); m.p. 82 °C; $^1$H NMR (300 MHz, $CDCl_3$) δ 6.94 (d, *J* = 8.0 Hz, 1H), 7.05 (t, *J* = 8.0 Hz, 1H), 7.37 (t, *J* = 8.0 Hz, 1H), 7.48 (d, *J* = 8.0 Hz, 1H) ppm; $^{13}$C NMR (75 MHz, $CDCl_3$) δ 113.3, 118.4, 120.6, 121.2, 124.4, 128.2, 131.7, 155.4 ppm; $^{19}$F NMR (282.4 MHz, $CDCl_3$) δ −75.5 ppm; GC/MS: $t_R$ = 5.45 min. (EI) *m*/*z*: 260, 191, 145, 121, 95, 69, 51.

**General Procedure for the synthesis of diols 6, 7 and 8**

4.7 g (30.5 mmol) biphenyl was diluted in 11 mL (74.0 mmol) *N*,*N*,*N*',*N*'-tetramethylethylenediamine (TMEDA). To this solution 45 mL (74.0 mmol, 1.6 M in hexane) *n*-BuLi was added dropwise by syringe and stirred for 15 h at rt. The resulting red-brown solution was stored at −20 °C. The formed crystals, 4.95 g (12.42 mmol) 2,2´-dilithiobiphenyl·2 TMEDA (M = 398.5 g/mol) were diluted in 150 mL diethyl ether and, at 0 °C, 25.0 mmol of the corresponding carbonyl compound was added. The reaction mixture was allowed to warm to rt and stirred for 24 h. Aqueous $NH_4Cl$-solution was added. The organic phase was separated and the aqueous phase extracted three times with ether. The combined organic phases were dried over $MgSO_4$ and concentrated under reduced pressure. The crude product was recrystallized from acetone to give the diols as colorless crystals.

**Analytic and spectroscopic data for 6:** Yield 3.4 g (9.2 mmol, 37%); m.p. 210 °C; $^1$H NMR (300 MHz, $CDCl_3$) δ 3.73 (s, 2H), 5.69 (s, 2H), 7.27–7.38 (m, 18H) ppm; $^{13}$C NMR (75 MHz, $CDCl_3$) δ 71.7, 125.8, 127.0, 127.4, 128.1, 128.3, 129.5, 140.0, 142.4, 143.1 ppm; GC/MS: $t_R$ = 12.65 min. (EI) *m*/*z*: 271, 257, 241, 215, 181, 165, 151, 105, 91, 77, 51.

**X-ray crystal data:** $C_{26}H_{22}O_2$; $M_r$ = 366.44; space group P-1; triclinic; a = 9.1578(6) Å, b = 9.5970(5) Å, c = 11.3407(6) Å, β = 83.794(3)°; V = 957.63(9) Å$^3$; Z = 2; T =

100(2) K; reflection total: unique: 4174, observed: 2785 (I>2σ(I)); R1= 0.0442, *w*R2 = 0.0966.

**Analytic and spectroscopic data for 7:** Yield 3.9 g (8.3 mmol, 33%); m.p. 217 °C; $^{1}H$ NMR (300 MHz, $CDCl_3$) δ 3.58 (s, 2H), 5.71 (s, 2H), 7.27–7.52 (m, 22H) ppm; $^{13}C$ NMR (75 MHz, $CDCl_3$) δ 71.7, 126.0, 127.0, 127.0, 127.4, 128.1, 128.4, 129.5, 130.1, 131.21, 133.6, 140.1, 142.4, 143.2 ppm.

**X-ray crystal data:** $C_{34}H_{26}O_2$; $M_r$ = 466.55; space group P-1; triclinic; a = 9.5302(5) Å, b = 10.1369(8) Å, c = 13.6192(9) Å, β = 71.831(3)°; V = 1364.4(4) $Å^3$; Z = 2; T = 446(2) K; reflection total:, unique: 4919, observed: 3350 (I>2σ(I)); R1= 0.0733, *w*R2 = 0.1006; GOF = 0.986

**Analytic and spectroscopic data for 8:** Yield 3.2 g (6.4 mmol, 26%); m.p. 198 °C; $^{1}H$ NMR (300 MHz, $CDCl_3$) δ 4.53 (s, 2H), 5.74 (d, *J* = 7.6 Hz, 2H), 6.78 (t, *J* = 8.0 Hz, 2H), 7.10 (d, *J* = 7.6 Hz, 2H), 7.23–7.35 (m, 8H), 7.81 (d, *J* = 8.0 Hz, 2H) ppm; $^{13}C$ NMR (75 MHz, $CDCl_3$) δ 123.0, 126.7, 126.5, 126.8, 127.0, 127.1, 127.8, 128.4, 135.1, 138.7, 140.3 ppm; $^{19}F$ NMR (282.4 MHz, $CDCl_3$) δ −74.04 ppm; GC/MS: $t_R$ = 11.48 min; (EI) *m*/*z*: 433, 239, 181, 152, 127, 105, 77, 51.

**General Procedure for the synthesis of phosphonates 9–15**

The diol and triethylamine (3.0 equiv) were diluted in dry toluene at 0 °C. To this solution $PCl_3$ (1.0 equiv) was added dropwise by syringe. The reaction was stirred at the same temperature for 2 h. To the resulting solution was added triethylamine (1.0 equiv) and $H_2O$ (1.0 equiv) at 0 °C. The reaction mixture was allowed to warm to rt and stirred for 1 h. Solid triethylammonium chloride was removed by filtration through a pad of $MgSO_4$ and the solution was concentrated under reduced pressure.

**Analytic and spectroscopic data for 9:** The crude product was recrystallized from *c*-hexane and toluene. Yield 0.05 g (0.3 mmol, 8%); $^{1}H$ NMR (300 MHz, $CDCl_3$)

δ 5.18 (s, 4H), 7.03 (d, *J* = 705.3 Hz, 1H), 7.28–737 (m, 4H) ppm; $^{31}$P NMR ($CDCl_3$) δ 13.08 (dquin, *J* = 705.3, *J* = 16.6 Hz, 1P) ppm.

**Analytic and spectroscopic data for 10:** The crude product was recrystallized from *c*-hexane and toluene. Yield 0.11 g (0.3 mmol, 18%); $^{1}$H NMR (300 MHz, $CDCl_3$) δ 5.20 (t, 1H), 7.31 (d, *J* = 755.0 Hz, 1H), 7.43–7.59 (m, 4H) ppm; $^{31}$P NMR ($CDCl_3$) δ 4.08 (dtq, *J* = 755.0, *J* = 16.8, *J* = 2.9 Hz, 1P) ppm; $^{19}$F NMR (282.4 MHz, $CDCl_3$) δ −75.9 ppm.

**Analytic and spectroscopic data for 11:** The crude product was recrystallized from toluene. Yield 0.11 g (0.4 mmol, 17%); $^{1}$H NMR (300 MHz, $CDCl_3$) δ 7.00 (d, *J* = 720.0 Hz, 1H), 5.24 (s, 4H) ppm; $^{31}$P NMR ($CDCl_3$) δ 13.90 (d, *J* = 720.0 Hz, 1P) ppm; $^{19}$F NMR (282.4 MHz, $CDCl_3$) δ −141.10, −153.81 ppm.

**Analytic and spectroscopic data for 12:** The crude product was recrystallized from *c*-hexane and toluene. Yield 0.03 g (0.17 mmol, 6%); $^{1}$H NMR (300 MHz, $CDCl_3$) δ 5.47 (s, 2H), 7.22 (d, *J* = 723.0 Hz, 1H), 7.08–7.48 (m, 4H) ppm; $^{31}$P-NMR ($CDCl_3$) δ −1.42 (d, *J* = 723.0 Hz, 1P) ppm.

**Analytic and spectroscopic data for 13:** The crude product was recrystallized from *c*-hexane and toluene. Yield 0.03 g (0.09 mmol, 5%); $^{1}$H NMR (300 MHz, $CDCl_3$) δ 7.35 (d, *J* = 758.0 Hz, 1H), 7.13–7.44 (m, 4H) ppm; $^{31}$P NMR ($CDCl_3$) δ −8.89 (d, *J* = 758.0 Hz, 1P) ppm; $^{19}$F NMR (282.4 MHz, $CDCl_3$) δ −74.3 ppm.

**Analytic and spectroscopic data for 14:** The crude product was purified by flash column chromatography on silica gel with hexane/ethylacetate as eluent. Yield 0.9 g (1.7 mmol, 81%); m.p. 213 °C; $^{1}$H NMR (300 MHz, $CDCl_3$) δ 5.62 (s, 2H), 7.13 (d, *J* = 713.6 Hz, 1H), 7.32–7.59 (m, 22H) ppm; $^{13}$C NMR (75 MHz, $CDCl_3$) δ 73.1, 122.5, 124.7, 125.1, 128.0, 128.7, 129.9, 130.3, 132.8, 136.8, 138.0, 138.5 ppm; $^{31}$P NMR ($CDCl_3$) δ 8.18 (d, *J* = 713.6 Hz, 1P) ppm.

**Analytic and spectroscopic data for 15:** The crude product was purified by flash column chromatography on silica gel with hexane/ethylacetate as eluent. Yield 0.7 g (1.2 mmol, 63%); m.p. 250 °C; $^1$H NMR (300 MHz, $CDCl_3$) δ 6.68–7.33 (m, 6H), 7.29 (d, *J* = 754.7 Hz, 1H), 7.73–7.81 (m, 2H) ppm; $^{13}$C NMR (75 MHz, $CDCl_3$) δ 26.9, 121.0, 122.0, 126.0, 126.6, 126.8, 127.0, 127.6, 127.7, 128.0, 128.3, 128.8, 131.4, 132.5, 132.8, 137.4, 136.7, 139.3, 140.5 ppm; $^{31}$P NMR ($CDCl_3$) δ −4.60 (d, *J* = 754.7 Hz, 1P) ppm; $^{19}$F NMR (282.4 MHz, $CDCl_3$) δ −70.82 ppm.

**X-ray crystal data:** $C_{28}H_{19}F_6O_3P$; $M_r$ = 548.40; space group $P2_1/n$; monoclinic; a = 10.3226(5) Å, b = 13.7172(9) Å, c = 16.8280(8) Å, β = 97.143(3)°; V = 2364.3(2) $Å^3$; Z = 4; T = 100(2) K; reflection total:, unique: 5153, observed: 3308 ($I>2\sigma(I)$); R1= 0.0610, *w*R2 = 0.1394; GOF = 1.056.

**General procedure for the reaction of acylsilanes with benzaldehyde**

Under argon, acylsilane (1.0 equiv), benzaldehyde (1.5 equiv) and phosphonate (10 mol %) were diluted in dry THF. At 0 °C, *n*-BuLi (40 mol %) was added dropwise by syringe. The reaction mixture was stirred at 0 °C for 1 h and for a further 30 min at rt. The solvent was removed under reduced pressure. A 1:1 MeOH/1 M HCl aqueous solution was added, the mixture was stirred for 10 min at rt, and then ether was added. The organic phase was separated and the aqueous phase extracted with ether three times. The combined organic phases were dried over $Na_2SO_4$ and concentrated under reduced pressure. The crude product was purified by flash column chromatography on silica gel with hexane/EtOAc, to afford the pure benzoin product.

**Crystallographic data:** The X-ray crystal data can be obtained free of charge from The Cambridge Crystallographic Data Centre through the following link: www.ccdc.cam.ac.uk/data_request/cif: CCDC-827239 (**5**), CCDC-827237 (**6**), CCDC-827238 (**14**). The structures were solved by direct methods (SHELXS [1,2]). O–H and

P–H in the X-ray crystal data were located in a difference Fourier map and refined isotropically. All other hydrogen atoms were positioned geometrically (C–H = 0.93–0.96 Å) and refined using a riding model with Uiso (H) = 1.2Ueq (C) and 1.5Ueq (methyl C).

## Computational details

The computations were carried out with GAUSSIAN03 [3]. B3LYP/6-31G(d) optimizations of the diols and phosphonate were performed in the gas phase [4-9] and all stationary points were characterized by frequency calculation. Zero point energies were scaled by 0.9806 [10].

## References

1. Sheldrick, G. M. *Acta Cryst.* **2008**, *64*, 112–122.

2. Spek, A. L. *Acta Cryst.* **2009**, *65*, 148–155.

3. Frisch, M. J.; Trucks, G. W.; Schlegel, H. B.; Scuseria, G. E.; Robb, M. A.; Cheeseman, J. R.; Montgomery, J. A.; Vreven, Jr.; Kudin, T. K. N.; Burant, J. C.; Millam, J. M.; Iyengar, S. S.; Tomasi, J.; Barone, V.; Mennucci, B.; Cossi, M.; Scalmani, G.; Rega, N.; Petersson, G. A.; Nakatsuji, H.; Hada, M.; Ehara, M.; Toyota, K.; Fukuda, R.; Hasegawa, J.; Ishida, M.; Nakajima, T.; Honda, Y.; Kitao, O.; Nakai, H.; Klene, M.; Li, X.; Knox, J. E.; Hratchian, H. P.; Cross, J. B.; Adamo, C.; Jaramillo, J.; Gomperts, R.; Stratmann, R. E.; Yazyev, O.; Austin, A. J.; Cammi, R.; Pomelli, C.; Ochterski, J. W.; Ayala, P.; Ayala, Y.; Morokuma, K.; Voth, G. A.; Salvador, P.; Dannenberg, J. J.; Zakrzewski, V. G.; Dapprich, S.; Daniels, A. D.; Strain, M. C.; Farkas, O.; Malick, D. K.; Rabuck, A. D.; Raghavachari, K. J.; Foresman, B.; Ortiz, J. V.; Cui, Q.; Baboul, A. G.; Clifford, S.; Cioslowski, J.; Stefanov, B. B.; Liu, G.;

Liashenko, A.; Piskorz, P.; Komaromi, I.; Martin, R. L.; Fox, D. J.; Keith, T. M.; Al-Laham, A. C.; Peng, Y.; Nanayakkara, A.; Challacombe, M.; Gill, P. M. W.; Johnson, B.; Chen, W.; Wong, M. W.; Gonzalez, C. and . Pople, J. A; Gaussian, Inc., Wallingford CT, **2004**.

4. Ditchfield, R. W.; Hehre, J.; Pople, J. A. *J. Chem. Phys.* **1971**, *54*, 724-729.

5. Rassolov, V. A.; Ratner, M. A.; Pople, J. A.; Redfern, P. C.; Curtiss, L. A. *J. Comp. Chem.* **2001**, *22*, 976–984.

6. Becke, A. D.; *J. Chem. Phys.* **1993**, *98*, 5648–5653. Implementation.

7. Stephens, P. J.; Devlin, J.; Chabalowski, C. F.; Frisch, M. J. *J. Phys. Chem.* **1994**, *98*, 11623–11627.

8. Lee, C.; Yang, W.; Parr, R. G. *Phys. Rev. B.* **1988**, *37*, 785–791.

9. Miehlich, B.; Savin, A.; Stoll, H.; Preuss, H. *Chem. Phys. Lett.* **1989**, *157*, 200–205.

10. Scott, A. P.; Radom, L. *J. Phys. Chem.* **1996**, *100*, 16502–16513.
